# Why the COVID-19 pandemic should be a call for action to advance equitable access to medicines

**DOI:** 10.1186/s12916-020-01661-3

**Published:** 2020-06-25

**Authors:** Jillian C. Kohler, Tim K. Mackey

**Affiliations:** 1 Leslie Dan Faculty of Pharmacy and Dalla Lana School of Public Health, University of Toronto, Toronto, Canada; 2 WHO Collaborating Center for Governance, Accountability and Transparency in the Pharmaceutical Sector and Professor, Leslie Dan Faculty of Pharmacy, University of Toronto, Toronto, Canada; 3 Department of Anesthesiology and Division of Infectious Diseases and Global Public Health, University of California, San Diego School of Medicine, San Diego, CA USA; 4 Department of Healthcare Research and Policy, University of California, San Diego - Extension, San Diego, USA; 5 Global Health Policy Institute, San Diego, CA USA

**Keywords:** Health policy, COVID-19, Access to medicines, Pharmaceutical policy, Pandemic response

## Background

With the COVID-19 pandemic, globally, we now face unprecedented challenges to access essential medicines and other health commodities. Drug shortages, hoarding of medicines and supplies, and the circulation of falsified health products have already exacerbated this once in a century global health challenge [1].

Pre-COVID-19, it was already estimated that 2 billion people lacked regular access to essential medicines [2]. Improving this gap is a long-standing global public health priority, associated with achieving Universal Health Coverage, a target in the United Nations’ Sustainable Development Goals. Despite decades of donor funding and international programs, availability of essential medicines remains wanting. High prices, lack of coverage, poor purchasing and distribution, uncertain product quality, inappropriate prescribing, and corruption are but some of the reasons why availability is undermined, whether it be in the United States or Malawi [3].

In anticipation of vital products to treat and prevent COVID-19, policy decisions about how to secure equitable access and affordability of these products globally need to happen in earnest. Simple questions about who will get access when a COVID-19 vaccine is developed, the price, how it will be paid for, who manufactures and distributes it, and ensuring the integrity of these products is protected from fake or substandard versions need meaningful deliberation now. Hence, the international debate about access to medicines has to be accelerated given the real-world needs of millions currently afflicted by COVID-19 and countless others who will in the future.

In the past, the importance of securing access to medicines for global health challenges, such as HIV/AIDS and hepatitis C virus, has been made abundantly and painfully clear [4, 5]. The basic questions of who gets access to what therapy, how, and when have been the subject of fierce debate and advocacy. Overlaying these questions is the fact that travel bans, trade-related retaliation, abuse of intellectual property (IP) rights, and other forms of protectionism have and may further limit supplies of needed therapies [6]. In fact, many countries have already banned or significantly limited export of protective equipment, medical devices, or medicines related to COVID-19 [7].

As a consequence of legitimate supply chains being blocked, the risk of falsified and substandard medicines grows [1]. Evidencing this risk, the European Medicines Agency and the World Health Organization (WHO) have issued warnings about the increase of falsified medicines and testing kits claiming to prevent, detect, treat, or cure COVID-19 [8, 9]. This includes online frauds involving the sales of suspect and unapproved products on e-commerce platforms, social media, and illegal online pharmacies [1]. Yet, the risk of falsified medicines is not limited only to products that may treat COVID-19, but extends to other drugs where shortages will persist [7].

## Call to action?

The world is waiting with tremendous hope and urgency for therapies and vaccines to treat and/or prevent COVID-19. The strong pressure on the pharmaceutical industry may very well incentivize a company or drug regulatory agency to rush an immature product to market. We need to be circumspect that in the urgent quest for life-saving therapies, existing regulatory safeguards put in place to ensure safety and efficacy are not bypassed. These concerns are even more acute with recent retractions of studies from *The New England Journal of Medicine* and *The Lancet* involving the antimalarial drug hydroxychloroquine, and announcement by the WHO that drug trials would be halted due to safety concerns [10].

There is also a need to raise and manage legitimate concerns about the power and influence of pharmaceutical companies and the potential for systematic bias within the R&D process. This raises questions of whether during grave public health emergencies, decisions about the research and development and access to essential and potentially life-saving treatments should be left to laissez-faire economic principles. There is a clear conflict of interest when relying on private companies to solve public health needs. Companies, by their nature, are required to bring value to their shareholders, but this approach may be flawed at normal times and decidedly more dangerous during health crises when the provision of public goods is needed.

Positively, we are seeing a shift in the speed of innovation and level of collaboration to stop this pandemic. This includes greater cooperation between governments, researchers, and with and within the life sciences industry. For example, Gilead Sciences recently announced deals with generic companies to manufacture and distribute experimental drug remdesivir via non-exclusive voluntary licenses. Less positively, nationalism, policy fragmentation, and the shutting down of borders may pose risks to the rapidity and reach of future treatments as countries turn increasingly inward [7]. Hence, now is the time to raise the urgency of ensuring equitable access to medicines to a supranational body, one focused on ensuring that there is a system in place to coordinate rational selection, procurement, access, distribution, and use across all countries before it is too late.

## Conclusion

Fortunately, the framework for this approach has already been put in place with international organizations, such as UNITAID, creating models to better ensure access to HIV, tuberculosis, and malaria medications; the Medicines Patent Pool which helps lower costs of medicines through voluntary licensing of IP; and the UN High-level Panel on Access to Medicines making a series of recommendations on how to promote and advance health technology and access.

More recently, the WHO announced the launch of the COVID-19 Access Pool, an international project to voluntarily share IP, scientific data, and health technology-related knowledge to fight COVID-19. The UN General Assembly also adopted a resolution calling for international cooperation to ensure global access to medicines, vaccines, and medical equipment for COVID-19, and this year’s World Health Assembly included a European Union-led resolution calling for “universal, timely and equitable access” to COVID-19 treatments.

These past and current actions should act as a catalyst for a global call to action to improve access to COVID-19 treatment, vaccine, and other countermeasures. It should also form the foundation for bolder international action to establish a global access to medicines regime charged with ensuring that no one gets left behind and that anyone who requires essential treatment for COVID-19 or other health maladies will get it.

## Data Availability

Not applicable

## Abbreviations

IP: Intellectual property
R&D: Research and development
WHO: World Health Organization
